# Proton-Conducting Polymer-Coated Carbon Nanofiber Mats for Pt-Anodes of High-Temperature Polymer-Electrolyte Membrane Fuel Cell

**DOI:** 10.3390/membranes13050479

**Published:** 2023-04-29

**Authors:** Kirill M. Skupov, Igor I. Ponomarev, Elizaveta S. Vtyurina, Yulia A. Volkova, Ivan I. Ponomarev, Olga M. Zhigalina, Dmitry N. Khmelenin, Evgeny N. Cherkovskiy, Alexander D. Modestov

**Affiliations:** 1 A.N. Nesmeyanov Institute of Organoelement Compounds of Russian Academy of Sciences, Vavilova St. 28, bld. 1, 119334 Moscow, Russia; gagapon@ineos.ac.ru (I.I.P.); ves1809@yandex.ru (E.S.V.); yvolk@ineos.ac.ru (Y.A.V.); ivan.ponomarev84@gmail.com (I.I.P.); 2 A.V. Shubnikov Institute of Crystallography of Federal Scientific Research Centre “Crystallography and Photonics” of Russian Academy of Sciences, Leninsky Av. 59, 119333 Moscow, Russia; zhigal@crys.ras.ru (O.M.Z.); xorrunn@gmail.com (D.N.K.); ev.cherkovskiy@gmail.com (E.N.C.); 3 A.N. Frumkin Institute of Physical Chemistry and Electrochemistry of Russian Academy of Sciences, Leninsky Av. 31, bld. 4., 119071 Moscow, Russia; amodestov@mail.ru

**Keywords:** carbon nanofibers, HT-PEMFC, proton-conducting polymer, platinum deposition, Pt/CNF, electrospinning, PIM-1, gas diffusion electrode, polyacrylonitrile, PBI-OPhT-P

## Abstract

High-temperature polymer-electrolyte membrane fuel cells (HT-PEM FC) are a very important type of fuel cell since they operate at 150–200 °C, allowing the use of hydrogen contaminated with CO. However, the need to improve stability and other properties of gas diffusion electrodes still hinders their distribution. Anodes based on a mat (self-supporting entire non-woven nanofiber material) of carbon nanofibers (CNF) were prepared by the electrospinning method from a polyacrylonitrile solution followed by thermal stabilization and pyrolysis of the mat. To improve their proton conductivity, Zr salt was introduced into the electrospinning solution. As a result, after subsequent deposition of Pt-nanoparticles, Zr-containing composite anodes were obtained. To improve the proton conductivity of the nanofiber surface of the composite anode and reach HT-PEMFC better performance, dilute solutions of Nafion^®^, a polymer of intrinsic microporosity (PIM-1) and N-ethyl phosphonated polybenzimidazole (PBI-OPhT-P) were used to coat the CNF surface for the first time. These anodes were studied by electron microscopy and tested in membrane-electrode assembly for H_2_/air HT-PEMFC. The use of CNF anodes coated with PBI-OPhT-P has been shown to improve the HT-PEMFC performance.

## 1. Introduction

The search for new and the development of existing electrocatalytic materials are very important for progress in the field of alternative energy, particularly fuel cells (FC) [1,2]. In the context of global decarbonization, renewable energy sources, compared with intermittent and unstable power generation, may eliminate the temporal and spatial gap between energy consumption by end-users and its availability; fuel cells can generate power flexibly at any time, as long as hydrogen supply is sufficient [3,4]. The high-temperature polymer-electrolyte membrane (HT-PEM) fuel cells [5,6] (Figure S1) possess many advantages, the most significant of which is the ability to operate with CO-contaminated hydrogen [7,8]. Polybenzimidazole (PBI)-type membranes doped with phosphoric acid are used for this type of FC and are capable of conducting protons without moisture [9,10,11]. This allows the polymer-electrolyte membrane to operate at elevated temperatures of 150–200 °C [12,13]. However, to increase its efficiency, further improvement of the main components of the high-temperature polymer-electrolyte membrane fuel cell (electrodes, membrane) is required [1,2,5,6,7,8,9,10,11,12,13,14,15,16,17]. 

Electrodes for HT-PEM FC are usually based on carbon black with Pt nanoparticles deposited as a catalyst. Due to their instability during the FC operation in phosphoric acid media at a sufficiently high temperature (150–200 °C) at high potential, their replacement with more stable carbon nanostructured materials is required. Recently, we have shown that carbon nanofiber (CNF) mats (self-supporting entire non-woven carbon nanofiber material) based on polyacrylonitrile (PAN) or polyheteroarylenes can be used as electrocatalyst support for Pt nanoparticles and applied as electrodes for the high-temperature polymer-electrolyte membrane FC [18,19,20,21,22,23,24,25,26,27,28,29,30,31,32,33]. 

The electrospinning method allows for obtaining sub-micrometer-sized polymer nanofibers [34], providing a higher nanofiber surface. The electrodes based on CNF were obtained using the method of electrospinning from the polymer solution [35,36], followed by temperature stabilization [37,38], pyrolysis [39,40] and Pt deposition. However, the electrocatalysts may require further optimization.

Triple point formation in Pt/C electrodes is essential for HT-PEM FC operation. It requires a carbon solid phase for electron transfer, a phosphoric acid medium for proton transfer and gas transport channels (hydrogen, oxygen or air) to be linked to the Pt electrocatalyst. Thereby, among the various factors affecting the electrode operation, the organization of continuous proton transport along the surface of the nanofibers is mostly required to enhance the transport of the proton from the triple point to the PBI membrane on the anode side of the fuel cell. Earlier, we showed that Zr-containing electrodes based on CNF mats are efficient in HT-PEM FC [22,23]. These CNF were obtained by pyrolysis at 1000 °C, leading to partial graphitization of the nanofibers [41,42], providing electrical conductivity.

Deposition of various polymers capable of protonation on carbon nanofibers of the FC electrode may improve the proton conductivity in electrode and FC performance. For instance, PIM-1 [43,44,45,46,47,48,49,50,51,52,53,54] is a microporous polymer with high hydrogen permeability. In addition, its protonation ability is well-known [55]. Similarly, Nafion^®^ [56,57,58,59,60] is a well-known proton-conducting polymer [2].

The already-mentioned PBI [61,62,63,64,65,66] are a large type of proton-conducting polymers which are used as polymer-electrolyte complexes with phosphoric acid for HT-PEM FC membranes. For example, the N-ethyl phosphonated PBI-type polymer (PBI-O-PhT-P), deposited on carbon nanofibers of the anode, introduces additional phosphonic functional groups, which may presumably improve proton conductivity. Earlier, we have shown that for a membrane based on PBI-O-PhT-P (also abbreviated as PEPBI-O-PH), when operated in HT-PEM FC, the value of the open circuit voltage was quite low (0.760 V) [67]. This can be explained by some features of the chemical structure of the polymer, particularly the loosening of polymer chains by side ethylphosphonic groups. As a result, the electrochemical crossover of hydrogen through the membrane is enhanced. 

The aim of this work is an attempt to enhance the HT-PEM FC performance by applying the proton-conducting polymers onto CNF; this can provide additional proton conductivity when applied onto the CNF, retaining hydrogen permeability under HT-PEMFC operation conditions.

In the current study, proton-conducting polymers PIM-1, PBI-O-PhT-P and Nafion^®^ were applied onto the PAN-based CNF and tested as Pt-anodes for high-temperature polymer-electrolyte membrane fuel cell for the first time.

## 2. Materials and Methods

### 2.1. Electrocatalyst Preparation

#### 2.1.1. Electrospinning

Composite PAN-based nanofibers were obtained by the needle-free electrospinning method from a free surface, according to the Nanospider^TM^ technology. The electrospinning process was performed on a NS Lab Nanospider^TM^ setup from Elmarco (Liberec, Czech Republic) at a relative humidity of 8%, at voltage of 69 kV, with a distance between electrodes of 190 mm from the electrospinning polymer solution, which contained 3.25 g of PAN (M_w_ 150·10^3^ Da), 0.1 g of UM-76 carbon black (~3 wt.% relative to PAN) and 0.03 g of zirconium (IV) chloride well-dispersed in 50 mL of N,N-dimethylformamide (DMF) in an ultrasonic bath for 3 h. As a result, PAN/UM/Zr composite nanofibers were obtained in the form of a mat.

#### 2.1.2. Stabilization, Zinc Deposition and Pyrolysis

PAN/UM/Zr nanofiber mat was stabilized (oxidized) at 350 °C in air for 2 h in a Binder MDL 115 heating chamber (Tuttlingen, Germany) to make the material suitable for further pyrolysis. The resulting material (PAN/UM/Zr/Ni-350) was immersed in 0.5 wt.% solution of zinc nitrate in water/ethanol (1:3 *v*/*v*) for ~24 h and dried at 100 °C for 2 h. Then, the sample was pyrolyzed at 1000 °C for 2 h under vacuum at a heating rate of 3 °C min^−1^ using a Carbolite (CTF 12/80/700) vacuum oven (Hope Valley, UK) equipped with a Eurotherm 3216 series controller. As a result, the shape of the CNF mat (PAN/UM/Zr-350/Zn-1000) was saved.

#### 2.1.3. Polymer Deposition

**Figure 1:**
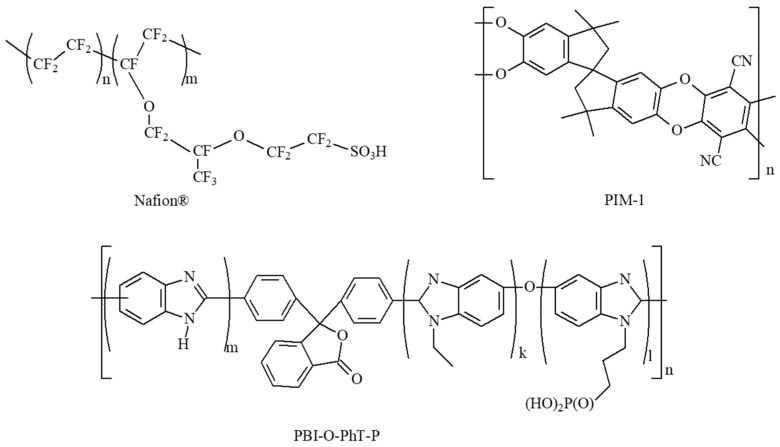
Chemical structures of proton-conducting polymers Nafion^®^, PIM-1 and PBI-O-PHT-P.

Nafion^®^ perfluorinated ion-exchange resin (a perfluorinated resin solution containing Nafion™ 1100 W, 5 wt.% in lower aliphatic alcohols and water, contains 15–20% water) was purchased from Sigma-Aldrich (St. Louis, MO, USA). PIM-1 was synthesized by the precipitation polyheterocyclization in dimethyl sulfoxide according to [68,69]. PBI-O-PhT-P (also abbreviated as PEPBI-O-PH) was synthesized according to [67]. The proton-conducting polymers PIM-1, PBI-O-PhT-P or Nafion^®^ (Figure 1) were deposited onto the CNF by adding 200 mg of 0.1 wt.% of polymer (PIM-1, PBI-O-PhT-P or Nafion^®^) solution in CH_2_Cl_2_, formic acid or water/isopropanol (1:1 *v/v*), respectively. As a result, polymer-coated CNF were obtained (PIM-1/CNF, PBI-O-PhT-P/CNF and Nafion/CNF, respectively).

#### 2.1.4. Platinum Deposition

Platinum was deposited onto the CNF and polymer-coated CNF mats, with a surface area of 6.76 cm^2^ separately for each mat, in 10 mL of aqueous solution containing the calculated amount of H_2_[PtCl_6_]·6H_2_O as a source of platinum and 0.5 g of formic acid as a reducing agent for 3 days to obtain Pt/CNF, Pt/PIM-1/CNF, Pt/PBI-O-PhT-P/CNF and Pt/Nafion/CNF electrocatalysts with a Pt concentration of 1.2 mg_Pt_ cm^−2^. The resulting electrocatalysts were thoroughly washed with distilled water and dried at 100 °C for 2 h under vacuum.

#### 2.1.5. Inverted Platinum and Polymer Deposition

For comparison, the proton-conducting polymers were applied to already platinated samples of Pt/CNF in the same way as described in Section 2.1.3. As a result, the polymer-coated electrocatalysts PIM-1/Pt/CNF, PBI-O-PhT-P/Pt/CNF and Nafion/Pt/CNF were also obtained.

#### 2.1.6. Elemental Analysis and Electrical Conductivity

The elemental analysis data were determined using an Elementar vario MICRO cube C,H,N-analyzer (Langenselbold, Germany) equipped with a thermal desorption column.

The in-plane electrical conductivities of the CNF mat (PAN/UM/Zr-350/Zn-1000) were found with a RLC E7-8 setup (Minsk, Belarus) equipped with a four-point probe. The sample thickness, which is necessary for the electrical conductivity measurements, was determined with an ElektroPhysik eXacto thickness gage (Cologne, Germany). The elemental analysis and electrical conductivity of the CNF are shown in Table 1.

### 2.2. Morphological Characterization

The structure of the composite еlectrospun polyacrylonitrile-based CNF was investigated by the methods of scanning electron microscopy (SEM) using a FEI Scios microscope (FEI, Hillsboro, OR, USA), transmission electron microscopy (TEM), high-resolution transmission electron microscopy (HR TEM), scanning transmission electron microscopy with a high-angle annular dark-field detector (HAADF STEM) and energy-dispersive X-ray spectroscopy (EDX) elemental mapping using a Thermo Fisher Scientific Osiris (Waltham, MA, USA) equipped with a high-angle annular dark field (HAADF) detector and Super-X EDX detection system based on Silicon Drift Detector (SDD) technology. Electron microscope images were analyzed using Digital Micrograph (GMS 3, Gatan, Pleasanton, CA, USA), TIA (TIA 16, Siemens AG, Munich, Germany), Esprit (Esprit 2, Bruker, Billerica, MA, USA) and JEMS software (P. Stadelmann JEMS—EMS Java version 2004 EPFL, Lausanne, Switzerland). For electron microscopy studies, the samples of CNF were well-dispersed in acetone to separate the fibers using an ultrasonic bath for 20–30 min. Then, the obtained suspensions were introduced onto copper lacey carbon grids.

### 2.3. HT-PEM Fuel Cell Operation

#### 2.3.1. Electrochemical Characterization

For the anode tests, the membrane-electrode assemblies (MEA) were prepared with a working area of 5 cm^2^. The MEA were placed in a standard Arbin Instruments testing cell (College Station, TX, USA) with two graphite flow field plates. The HT-PEM fuel cell was operated with a typical Celtec^®^-P 1000 MEA cathode [70] and the CNF-based anodes developed in this study. The PBI-OPhT membrane, cross-linked with zirconium acetylacetonate and doped with phosphoric acid (350–400%, up to 25 molecules of *o*-phosphoric acid per polymer unit), which was developed earlier by our group [71,72,73], was used for the MEA. Fuel cell operation was carried out at 160 and 180 °C. The anode was supplied with hydrogen obtained through electrolysis from a GVCh-6 hydrogen generator (Khimelektronika, Moscow, Russia) at a rate of 200 mL min^−1^ and the cathode was supplied with atmospheric air at a rate of 1000 mL min^−1^ without additional humidification. The polarization curves were obtained on a P-150X Potentiostat-galvanostat (Electrochemical Instruments, Chernogolovka, Russia). For voltammetry measurements, the fuel cell voltage was scanned at a rate of 10 mV s^−1^ in the cell voltage range 0.95–0.02 V.

The measurements of the HT-PEMFC membrane through plane resistance (mΩ cm^2^) were performed by the method of electrochemical impedance spectroscopy (EIS). The EIS experiments were performed on a SmartStat PS-250 Potentiostat-galvanostat (Electrochemical Instruments, Chernogolovka, Russia) at 0.4 A cm^−2^, applying a sinusoidal current with an amplitude of 50 mA in the frequency range 50 kHz–0.1 Hz.

#### 2.3.2. Hydrogen Crossover Measurements

Hydrogen crossover through the membrane at the operating temperatures of the HT-PEM fuel cell was measured by the method of linear sweep voltammetry by supplying hydrogen to the anode and argon (99.998% purity) to the cathode; the gases were supplied at an ambient pressure with flow rates of 50 mL min^−1^. Finally, the open-circuit voltage reached a steady-state value of about 120 mV, and the voltage was swept slowly at a rate of 1 mV s^−1^ to 500 mV; the current of hydrogen oxidation was recorded. Hydrogen penetrated the membrane, then appeared in the cathode catalyst layer and was completely oxidized. The current value measured under these conditions is assumed to be equal to the flow of hydrogen diffusing through the membrane. The current density corresponding to the crossover of hydrogen through the membrane was quantified at 350 mV. Higher electrode potentials were avoided to prevent the oxidation of platinum.

### 2.4. Adsorption Studies

#### 2.4.1. N_2_ Adsorption 

Nitrogen adsorption isotherms were obtained on a 3P Micro 200 Surface Area and Pore Size Analyzer (3P Instruments, Odelzhausen, Germany) at 77 K in the pressure range 10^−3^–1 bar. The Brunauer–Emmett–Teller (BET) equation was applied to the N_2_ adsorption isotherm data according to the Rouquerol criteria [74]. N_2_ cross-sectional area and adsorbed N_2_ density were taken as 0.162 nm^2^ and 0.808 g mL^−1^, respectively. 

#### 2.4.2. CO_2_ Adsorption 

CO_2_ adsorption isotherms [(adsorbed volume V_STP_ (cm^3^ g^−1^) calculated for standard temperature and pressure (STP) conditions (1 bar, 273 K) vs. relative pressure p/p_0_ (p_0_ is saturated vapor pressure)] were obtained on a 3P Micro 200 Surface Area and Pore Size Analyzer (3P Instruments, Odelzhausen, Germany) at 10^−3^–1 bar and 273 K. The Dubinin-Radushkevich (DR), non-local density functional theory (NL-DFT) and grand canonical Monte-Carlo (GCMC) methods were applied to the CO_2_ adsorption isotherm data using NovaWin, version 11.04, Quantachrome Instruments (Boynton Beach, FL, USA), considering a CO_2_ cross-sectional area of 0.210 nm^2^, adsorbed CO_2_ density of 1.044 g mL^−1^, saturated vapor pressure of the adsorbate p_0_ of 3.485 MPa and affinity coefficient β of 0.35 [75]. Pore-size distributions were obtained according to the NL-DFT method. 

## 3. Results

### 3.1. Electron Microscopy

Three proton-conducting polymers, PIM-1, PBI-O-PhT-P and Nafion^®^ (Figure 1), may provide additional proton conductivity when applied to the CNF. We expect this improvement because PIM-1 protonation ability is well-known [55]. Nafion^®^ [56,57,58,59,60] is another well-known proton-conducting polymer [2]. The N-ethyl phosphonated PBI-type polymer (PBI-O-PhT-P), deposited on the CNF of the anode, introduces additional phosphonic functional groups, which can improve proton conductivity. Earlier, we showed that a proton-conducting membrane can be prepared from PBI-O-PhT-P (also abbreviated as PEPBI-O-PH or PhEPBI-O-PhT) [67]; its proton conductivity was described in detail [76]. At the same time, it could remain permeable to hydrogen under the operation conditions of HT-PEMFC due to the peculiarities of its structure, as explained before. Platinum nanoparticles were deposited onto polymer-coated carbon nanofibers (polymer/CNF) to obtain electrodes of general formula Pt/polymer/CNF. The Pt/polymer/CNF structures were studied by the method of electron microscopy. 

**Figure 2:**
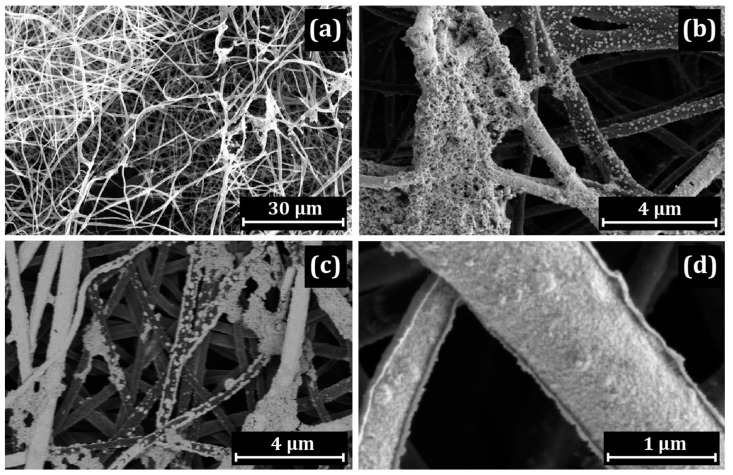
SEM images of Pt/PIM/CNF: (**a**) general view; (**b**–**d**) distribution of Pt across the mat thickness.

SEM images of the CNF structure with the deposited PIM-1 are shown in Figure 2.

Platinum was deposited on the PIM/CNF in an aqueous solution of H_2_[PtCl_6_], using HCOOH as a reducing agent. The general SEM image (Figure 2a) indicates that the fibers located on the surface of the mat are covered with a uniform layer of platinum. However, the CNF lying in the depth are covered with platinum “islands” or have no coating at all (Figure 2b,c). At the same time, porous accumulations of platinum several microns in size are observed on the CNF surface (Figure 2d). Most likely, they are located in a polymer shell. 

**Figure 3:**
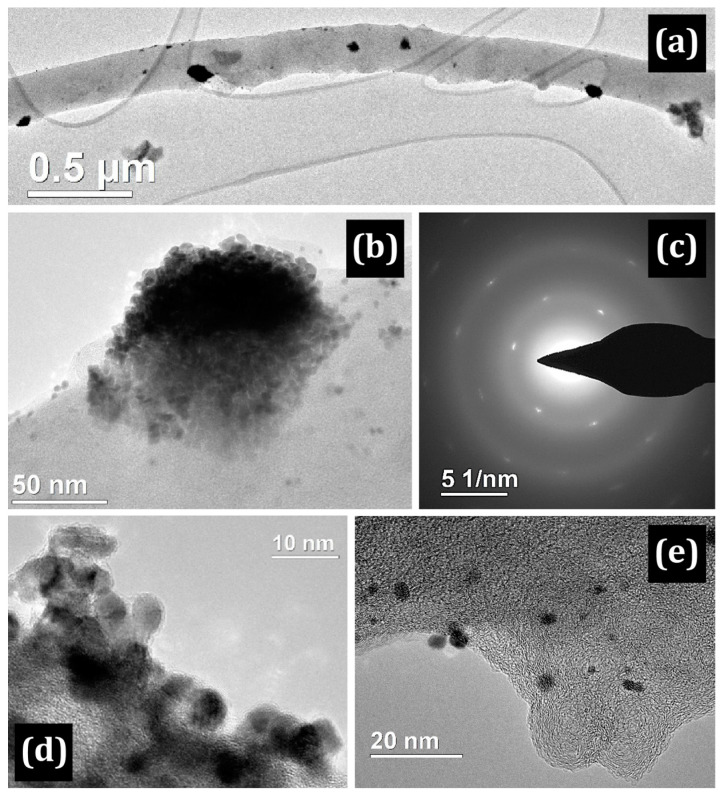
(**a**) TEM image of a Pt/PIM/CNF nanofiber; (**b**) Pt island on a PIM-1 shell of the CNF and (**c**) its SAED image with clear Pt signals; (**d**–**e**) HR TEM images of a fiber.

Figure 3 shows a bright field TEM image of a single Pt island that is “immersed” in the polymer. 

The presence of a polymer shell on the CNF is also indicated by a selected area electron diffraction (SAED) pattern with a strong background and individual bright signals of platinum (Figure 3a–c). The island of platinum is a polycrystalline agglomerate with individual nanocrystals of 5–10 nm. In addition, individual platinum particles of rounded shape with a size of 2–10 nm are clearly observed on the fibers.

**Figure 4:**
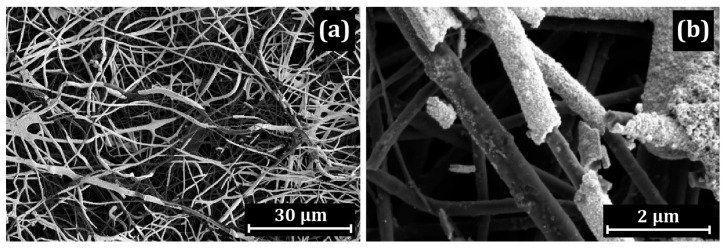
SEM images of Pt “skin” on the Pt/Nafion/CNF surface: (**a**) general view of the nanofibers; (**b**) separate nanofibers.

**Figure 5:**
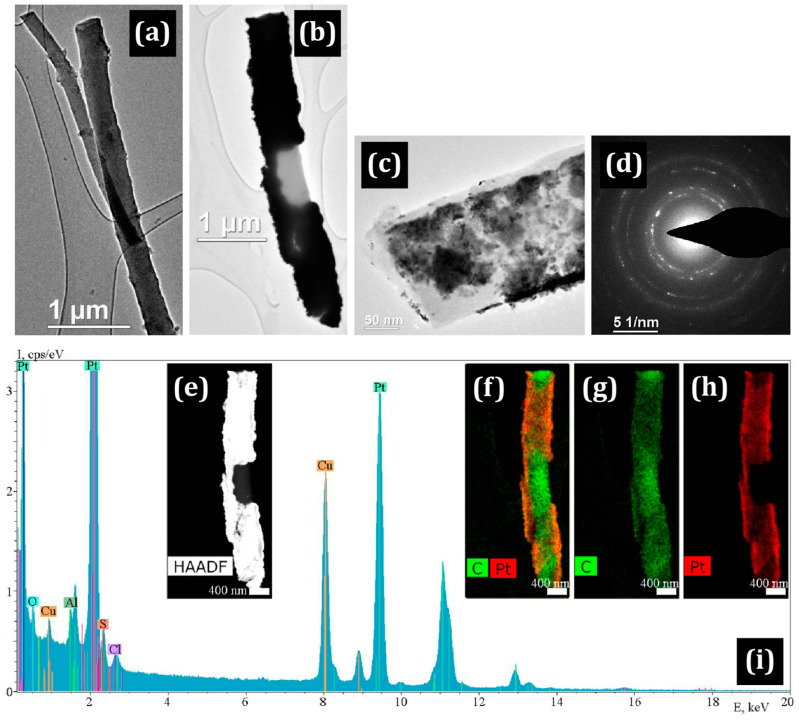
(**a**–**c**) TEM images of the Pt/Nafion/CNF; (**d**) the corresponding SAED pattern; (**e**) STEM HAADF image of carbon nanofiber, (**f**–**h**) elemental maps and (**i**) the corresponding EDX spectrum.

When Pt is deposited on Nafion/CNF, the formation of a Pt “skin” with a width of up to 120 nm is observed in the upper layers of the mat. Due to the large thickness, the coating peels off and breaks on many fibers, which is clearly seen in the TEM and SEM images (Figure 4 and Figure 5).

**Figure 6:**
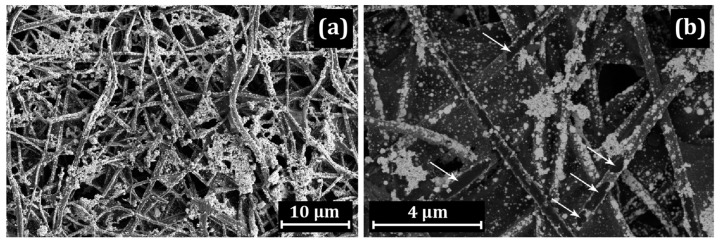
SEM images of Pt/PBI-OPhT-P/CNF: (**a**) general view of nanofibers and (**b**) Pt and polymer distribution on the CNF surface. Arrows show the places uncoated with polymer on the CNF surface.

**Figure 7:**
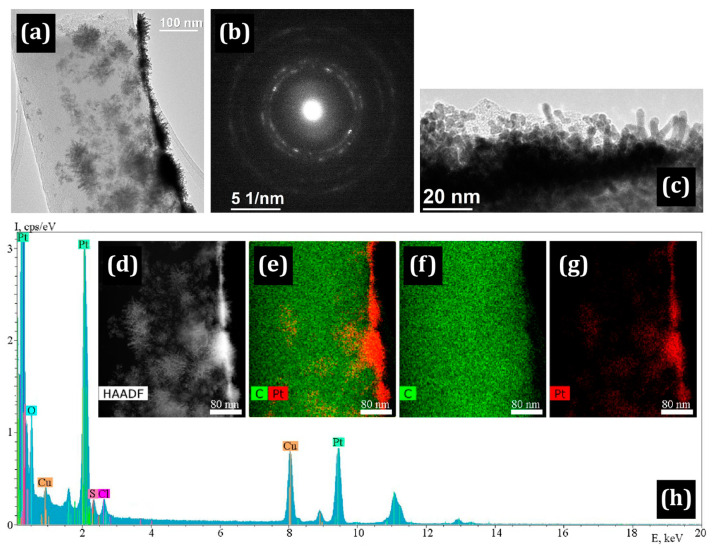
Needle-like Pt nanoparticles on the Pt/PBI-OPhT-P/CNF surface: (**a**) TEM image, (**b**) the corresponding SAED pattern; (**c**) HR TEM image and (**d**) the corresponding STEM HAADF image, elemental maps for (**e**) C and Pt, (**f**) C and (**g**) Pt; (**h**) the corresponding EDX spectrum.

In contrast to the Pt/PIM/CNF and Pt/Nafion/CNF samples, the polymer coating in the Pt/PBI-O-PhT-P/CNF sample is less uniformly distributed over the surface. It can be seen that the CNF are coated with the polymer, while the platinum possesses the shape of islands. In some places, the polymer layer is absent (shown by arrows in Figure 6b). TEM studies (Figure 7) show that the morphology of platinum particles differs from those indicated above: Pt nanocrystals possess an acicular shape. The length of some Pt needles can be up to 20 nm long. At the same time, particles of rounded shape are present. The needle-shaped particles placed directly on the nanofiber with the corresponding SAED image are shown in Figure 7a,b. The HAADF STEM image, element distribution maps and corresponding EDX spectrum illustrating the distribution of Pt along the length of the fiber are shown in Figure 7c–h.

**Figure 8:**
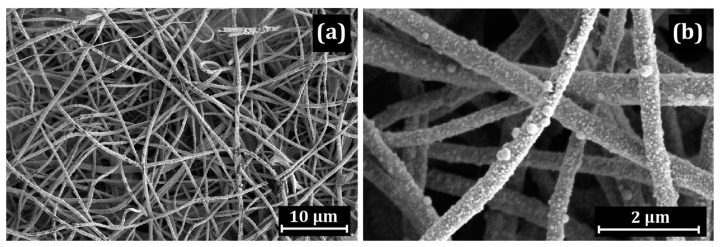
SEM images of the pristine polymer-uncoated Pt/CNF: (**a**) general view; (**b**) uniform distribution of Pt nanoparticles on the surface.

**Figure 9:**
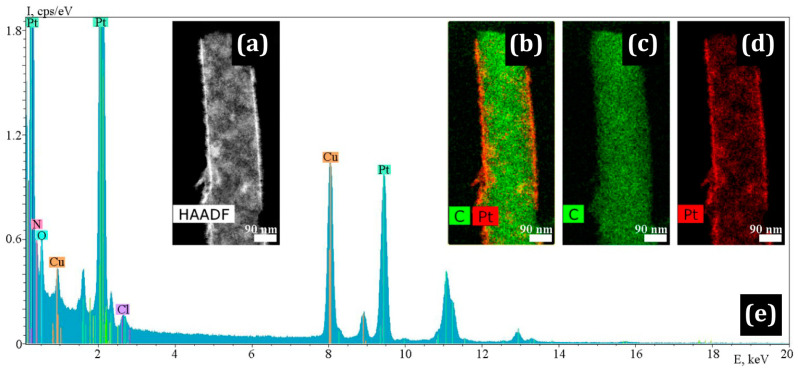
(**a**) STEM HAADF image of the pristine Pt/CNF; element distribution maps for (**b**) C and Pt, (**c**) C and (**d**) Pt; (**e**) the corresponding EDX spectrum.

Comparison with uncoated Pt/CNF (prepared without a polymer coating) showed that platinum is uniformly distributed on the fiber surface throughout the entire depth of the mat, with a predominance of the acicular morphology of platinum (Figure 8 and Figure 9).

TEM and SEM images of nanofibers with the deposited PBI-O-PhT-P are shown in Figure 6 and Figure 7. 

### 3.2. Adsorption Studies

The initial non-platinated CNF (PAN/UM/Zr-350/Zn-1000) sample was studied by the N_2_ and CO_2_ adsorption methods to determine its specific surface area and specific volume. The data from the Brunauer–Emmett–Teller (BET) method applied to the N_2_ adsorption isotherm (77 K) show quite low values (15 m^2^/g). Possibly, it is due to the inaccessibility of micropores to nitrogen due to kinetic difficulties at such a low temperature (77 K). Therefore, to overcome the kinetic difficulties, an alternative method of the CO_2_ adsorption (273 K) was applied [77]. The CO_2_ adsorption isotherm data were used to find the micropore specific volume (V), adsorption energy (E) and pore width (D) values by the DR method and the micropore specific volume (V) and specific surface area (S) values by the NL-DFT and GCMC methods (Table 2). 

**Figure 10:**
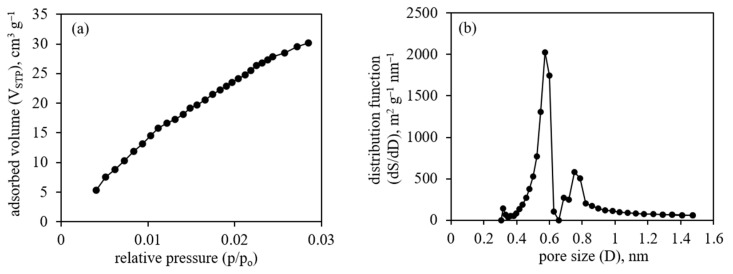
(**a**) CO_2_ adsorption isotherm at 273 K; (**b**) NL-DFT pore-size distribution for the CNF (PAN/UM/Zr-350/Zn-1000) sample.

The differences in values obtained by different methods are typical, since different theoretical approaches and considerations are applied to different methods. The adsorption isotherm and NL-DFT pore-size distribution data are shown in Figure 10. 

The presented data on microporosity (Table 2, Figure 10) are typical for microporous materials and suggest the possibility to use the material as a support for Pt nanoparticle catalyst in HT-PEM FC.

### 3.3. HT-PEM Fuel Cell Performance

**Figure 11:**
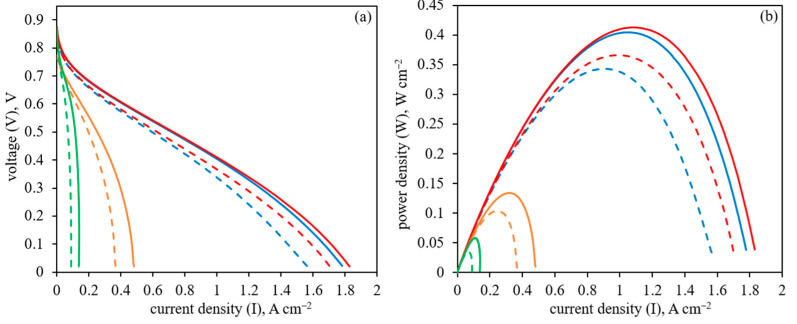
(**a**) Polarization curves and (**b**) power density data for HT-PEM FC MEA with CNF-based anodes at 180 °C for Pt/CNF (blue, solid line), Pt/PBI-O-PhT-P/CNF (red, solid line), Pt/PIM-1/CNF (orange, solid line), Pt/Nafion/CNF (green, solid line); and at 160 °C for Pt/CNF (blue, dashed line), Pt/PBI-O-PhT-P/CNF (red, dashed line), Pt/PIM-1/CNF (orange, dashed line), Pt/Nafion/CNF (green, dashed line).

The performance of the HT-PEM FC MEA with different Pt/polymer/CNF anodes when platinum is deposited on polymer-coated CNF is shown in Figure 11. 

The polarization curves and power density data for the Pt/polymer/CNF are compared with the Pt/CNF sample without polymer coating in the HT-PEM FC operation at 160 and 180 °C. As seen in Figure 10, Pt/PBI-O-PhT-P/CNF shows higher performance compared with Pt/CNF. At the same time, the application of CNF coated with Nafion^®^ and PIM-1 as supports for platinum nanoparticles leads to much lower HT-PEM FC performance. It can be related to a less uniform polymer coating on the CNF in the case of PBI-O-PhT-P compared with Nafion^®^ or PIM-1, as shown above by electron microscopy. As a result, more platinum nanoparticles possess an acicular shape and grow directly on the CNF surface instead of being located on the polymer layer, which improves electron transfer during HT-PEM FC operation.

**Figure 12:**
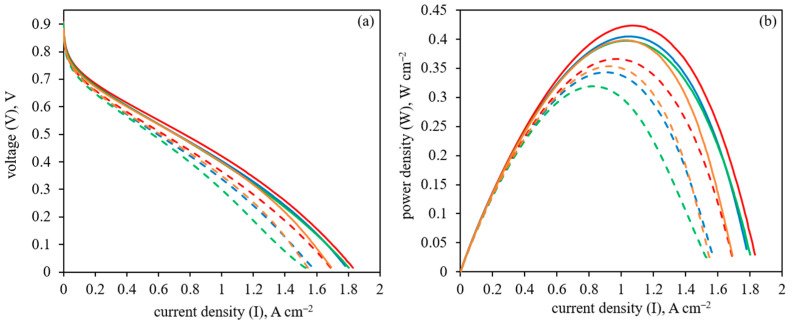
(**a**) Polarization curves and (**b**) power density data for HT-PEM FC MEA with CNF-based anodes at 180 °C for Pt/CNF (blue, solid line), PBI-O-PhT-P/Pt/CNF (red, solid line), PIM-1/Pt/CNF (orange, solid line), Nafion/Pt/CNF (green, solid line); and at 160 °C for Pt/CNF (blue, dashed line), PBI-O-PhT-P/Pt/CNF (red, dashed line), PIM-1/Pt/CNF (orange, dashed line), Nafion/Pt/CNF (green, dashed line).

The inverted anode structure, when the polymer was applied on the already prepared CNF with deposited platinum (Pt/CNF), was also studied. The polarization curves and power density data for these samples of general formula polymer/Pt/CNF for HT-PEM FC operation at 160 and 180 °C are shown in Figure 12.

As can be seen from Figure 12, the HT-PEMFC performance for the anode coated with PBI-O-PhT-P (PBI-O-PhT-P/Pt/CNF) is still higher than for the uncoated Pt/CNF sample. The application of Nafion^®^ and PIM-1 onto the already prepared Pt/CNF resulted in a very slight decrease in HT-PEMFC performance, making its performance similar to the uncoated Pt/CNF sample.

Data on the membrane resistance and hydrogen crossover through the membrane during HT-PEMFC operation at 160 and 180 °C, obtained by the electrochemical impedance spectroscopy and linear sweep voltammetry methods, are given in Table 3.

As can be seen from Table 3, the membrane resistance at 160 °C is in the range 60–90 mΩ cm^−2^ and, at 180 °C, is in the range 65–90 mΩ cm^−2^. These values are typical for the PBI-O-PhT membrane and do not differ significantly, since the same PBI-O-PhT membrane was used in all studied MEA. At the same time, the higher resistance for samples with Nafion^®^ (91.2 mΩ cm^−2^ for Pt/Nafion/CNF) may be related to a more uniform deposition of Nafion^®^ on the CNF surface, according to electron microscopy data, which slightly worsens the electrical conductivity. The hydrogen crossover through the membrane at 160 and 180 °C (Figure S2) corresponds to the typical PBI-O-PhT membrane operation in HT-PEMFC and does not exceed 3–5 mA cm^−2^. 

## 4. Discussion

In this part, a discussion of the main findings and the revealed general observations is provided for three proton-conducting polymers (Figure 1) applied on CNF (Table 1). The polymers applied onto the CNF are PIM-1 (Figure 2 and Figure 3), Nafion^®^ (Figure 4 and Figure 5) and PBI-O-PhT-P (Figure 6 and Figure 7). The data is compared with the pristine samples without polymer deposition (Figure 8 and Figure 9).

The application of proton-conducting polymers on carbon nanofiber materials in order to increase the proton conductivity of electrodes is possible in two ways. The first possibility is to apply the polymer onto the CNF before platinum deposition. In this case, platinum nanoparticles would be open to gases, but the electron transfer between platinum nanoparticles and carbon material may be disturbed. Such a situation can be observed using electron microscopy methods when platinum nanoparticles are “immersed” into a polymer layer. Another possibility is to deposit platinum nanoparticles directly onto the CNF, followed by the deposition of a proton-conducting polymer. In this case, electron transfer between platinum nanoparticles and carbon material can be ensured. At the same time, after polymer deposition, platinum can be excessively covered with the polymer, which may prevent good contact of platinum nanoparticles with gases, particularly hydrogen. One of the best ways to evaluate the effectiveness of the presented approach is to perform HT-PEM fuel cell tests where polymer-coated CNF with deposited Pt nanoparticles are used as electrodes, particularly anodes. The presented data for the microporosity studies (Figure 10, Table 2) are typical for microporous PAN-based CNF and suggest the possibility of using the material as a support for a Pt nanoparticle catalyst in HT-PEM FC.

According to the HT-PEM FC performance data presented in Figure 11, when Pt is deposited on polymer-coated CNF, the HT-PEM FC performance depends on the nature of the studied polymer. In the case of Nafion^®^ and PIM-1, the HT-PEM FC performance is significantly lower compared with the case of the uncoated Pt/CNF. At the same time, in the case of PBI-O-PhT-P, the performance is higher than in the case of the uncoated Pt/CNF. This can be explained by a less uniform PBI-O-PhT-P coating on the CNF, which leaves some parts of the CNF uncoated (as shown in Figure 6), compared with Nafion^®^ and PIM-1 coatings, which appear more uniform (Figure 2 and Figure 4). A more uniform polymer coating results in poorer contacts between Pt nanoparticles and CNF and, presumably, affects the distribution of Pt nanoparticles, which also looks irregular compared with the Pt/CNF sample (Figure 8 and Figure 9). A less uniform PBI-O-PhT-P coating on the CNF results in a significant number of places uncoated with polymer. It allows platinum nanoparticles to grow directly on the carbon surface of the CNF, however, very close to the proton-conducting polymer necessary for proton transfer. Apparently, it results in a better distribution of Pt nanoparticles and an acicular morphology of Pt nanoparticles, which has been shown in the past to be more active than spherical. The uncoated Pt/CNF also possesses a uniform distribution of platinum predominantly acicular in shape (Figure 8 and Figure 9). Obviously, these features, combined with higher proton transfer due to polymer deposition, can improve the HT-PEM FC performance for Pt/PBI-O-PhT-P/CNF, as seen from the data in Figure 11. Thus, the HT-PEMFC performance order depending on the polymer applied onto the anode is as follows:

PBI-O-PhT-P > polymer-uncoated > PIM-1 > Nafion^®^

The inverted method of polymer deposition, when the polymer is applied to the already Pt-decorated CNF, leads to a very different situation. Interestingly, the order of the HT-PEMFC performance depending on the polymer remains the same, but the values are much closer to each other and to the uncoated Pt/CNF sample. This can be explained by the Pt nanoparticles of acicular morphology already formed on the CNF surface with a uniform distribution, which were used for the proton-conducting polymer deposition. The application of PIM-1 and Nafion^®^ reduces the HT-PEM FC performance, slightly reducing H_2_ transport to platinum, but not significantly, compared to the uncoated Pt/CNF sample (Figure 12, Table 3). At the same time, the application of PBI-O-PhT-P onto Pt/CNF leads to an improvement in the HT-PEM FC performance, presumably due to better proton conductivity in the anode without preventing H_2_ transport to platinum nanoparticles (Figure 12, Table 3).

The electrochemical impedance spectroscopy and linear sweep voltammetry provide the membrane resistance and hydrogen gas crossover through the membrane values in the assembled HT-PEMFC at 160 and 180 °C. The values are similar and correspond to the typical PBI-O-PhT membrane. However, the slightly higher resistance values in the case of Nafion^®^ may reflect some disruption in electrical conductivity and result in inferior HT-PEM FC performance compared with other polymers and the uncoated sample. 

## 5. Conclusions

The study shows that HT-PEMFC performance depends on the proton-conducting polymer applied to the anode. The order of positive effect of the polymer on HT-PEM fuel cell performance is as follows:

PBI-O-PhT-P > polymer-uncoated sample > PIM-1 > Nafion^®^

This is true for both cases: direct deposition of a proton-conducting polymer onto the CNF followed by deposition of Pt nanoparticles and deposition of a polymer onto already prepared Pt-decorated CNF. However, for the second case of polymer deposition, the values are much closer to each other. The EIS data correspond to these findings. 

As shown by electron microscopy, the difference in performance can be explained by how the polymer coats the CNF and by platinum morphology. The obtained data show the advantage of the non-uniform coating of CNF by PBI-O-PhT-P.

## Figures and Tables

**Table 1:** Electrical conductivity and elemental analysis of pyrolyzed CNF (PAN/UM/Zr-350/Zn-1000).

Sample	σ, S/cm	%C	%N	%H	%Zr
CNF	24.7	82.9	5.24	1.47	0.2

**Table 2:** The specific volume (V), specific surface area (S), adsorption energy (E) and pore width (D) values of the micropores for the CNF (PAN/UM/Zr-350/Zn-1000) sample from the CO_2_ adsorption data (273 K) by the DR, NL-DFT and GCMC methods.

Sample	DR			NL-DFT		GCMC	
	V, cm^3^ g^−1^	E, kJ mol^−1^	D, nm	S, m^2^ g^−1^	V, cm^3^ g^−1^	S, m^2^ g^−1^	V, cm^3^ g^−1^
CNF	0.194	20.99	1.24	310	0.107	341	0.135

**Table 3:** Membrane resistance and hydrogen crossover through the membrane data for HT-PEM FC MEA with different anode samples.

Sample	R_memb_ (160 °C), mΩ cm^2^	R_memb_ (180 °C), mΩ cm^2^	I_H2 crossover_ (160 °C), mA cm^−2^	I_H2 crossover_ (180 °C), mA cm^−2^
Pt/CNF	59.0 ± 0.5	65.0 ± 0.5	4.8 ± 0.1	5.4 ± 0.1
Pt/PBI-O-PhT-P/CNF	61.0 ± 0.5	69.0 ± 0.5	1.1 ± 0.1	1.3 ± 0.1
Pt/PIM-1/CNF	n/d	n/d	n/d	n/d
Pt/Nafion/CNF	91.2 ± 0.5	90.5 ± 0.5	n/d	n/d
PBI-O-PhT-P/Pt/CNF	78.5 ± 0.5	76.0 ± 0.5	0.2 ± 0.1	0.3 ± 0.1
PIM-1/Pt/CNF	71.1 ± 0.5	68.5 ± 0.5	n/d	n/d
Nafion/Pt/CNF	82.0	80.0	0.2	0.4

n/d—not determined.

## Data Availability

The authors confirm that the data supporting the findings of this study are available within the article and its Supplementary Materials.

## Supplementary Materials

The following supporting information can be downloaded at: https://www.mdpi.com/article/10.3390/membranes13050479/s1, Figure S1: HT-PEMFC operation scheme; Figure S2: EIS Nyquist plots.

## References

[1] Li Q., Aili D., Hjuler H.A., Jensen J.O. (2016). High Temperature Polymer Electrolyte Membrane Fuel Cells, Approaches, Status and Perspectives.

[2] Zhang J. (2008). PEM Fuel Cell Electrocatalysts and Catalyst Layers, Fundamentals and Applications.

[3] Rosli R.E., Sulong A.B., Daud W.R.W., Zulkifley M.A., Husaini T., Rosli M.I., Majlan E.H., Haque M.A. (2017). A review of high-temperature proton exchange membrane fuel cell (HT-PEMFC) system. Int. J. Hydrogen Energy.

[4] Zhang H., Sun C., Ge M. (2022). Review of the Research Status of Cost-Effective Zinc–Iron Redox Flow Batteries. Batteries.

[5] Kalathil A., Raghavan A., Kandasubramanian B. (2019). Polymer Fuel Cell Based on Polybenzimidazole Membrane: A Review. Polym. Plast. Technol. Mater..

[6] Chandan A., Hattenberger M., El-Kharouf A., Du S., Dhir A., Self V., Pollet B.G., Ingram A., Bujalski W. (2013). High temperature (HT) polymer electrolyte membrane fuel cells (PEMFC)—A review. J. Power Sources.

[7] Araya S.S., Zhou F., Liso V., Sahlin S.L., Vang J.R., Thomas S., Gao X., Jeppesen C., Kær S.K. (2016). A comprehensive review of PBI-based high temperature PEM fuel cells. Int. J. Hydrogen Energy.

[8] Pingitore A.T., Molleo M., Schmidt T.J., Benicewicz B.C., Lipman T., Weber A. (2019). Polybenzimidazole Fuel Cell Technology: Theory, Performance, and Applications. Fuel Cells and Hydrogen Production. Encyclopedia of Sustainability Science and Technology Series.

[9] Zeis R. (2015). Materials and characterization techniques for high-temperature polymer electrolyte membrane fuel cells. Beilstein J. Nanotechnol..

[10] Authayanum S., Im-Orb K., Arpornwichnop A. (2015). A review of the development of high temperature proton exchange membrane fuel cells. Chin. J. Catal..

[11] Quartarone E., Angioni S., Mustarelli P. (2017). Polymer and Composite Membranes for Proton-Conducting, High-Temperature Fuel Cells: A Critical Review. Materials.

[12] Escorihuela J., Olvera-Mancilla J., Alexandrova L., del Castillo L.F., Compañ V. (2020). Recent Progress in the Development of Composite Membranes Based on Polybenzimidazole for High Temperature Proton Exchange Membrane (PEM) Fuel Cell Applications. Polymers.

[13] Myles T., Bonville L., Maric R. (2017). Catalyst, Membrane, Free Electrolyte Challenges, and Pathways to Resolutions in High Temperature Polymer Electrolyte Membrane Fuel Cells. Catalysts.

[14] Zhang J., Xie Z., Zhang J., Tang Y., Song C., Navessin T., Shi Z., Song D., Wang H., Wilkinson D.P. (2006). High temperature PEM fuel cells. J. Power Sources.

[15] Delikaya O., Bevilacqua N., Eifert L., Kunz U., Zeis R., Roth C. (2020). Porous electrospun carbon nanofibers network as an integrated electrode@gas diffusion layer for high temperature polymer electrolyte membrane fuel cells. Electrochim. Acta.

[16] Wang P., Li X., Liu Z., Peng J., Shi C., Li T., Yang J., Shan C., Hu W., Liu B. (2022). Construction of highly conductive PBI-based alloy membranes by incorporating PIMs with optimized molecular weights for high-temperature proton exchange membrane fuel cells. J. Membr. Sci..

[17] Guo Z., Perez-Page M., Chen J., Ji Z., Holmes S.M. (2021). Recent advances in phosphoric acid–based membranes for high–temperature proton exchange membrane fuel cells. J. Energy Chem..

[18] Skupov K.M., Vtyurina E.S., Ponomarev I.I., Ponomarev I.I., Aysin R.R. (2023). Prospective carbon nanofibers based on polymer of intrinsic microporosity (PIM-1): Pore structure regulation for higher carbon sequestration and renewable energy source applications. Polymer.

[19] Ponomarev I.I., Razorenov D.Y., Ponomarev I.I., Volkova Y.A., Skupov K.M., Lysova A.A., Yaroslavtsev A.B., Modestov A.D., Buzin M.I., Klemenkova Z.S. (2021). Polybenzimidazoles via polyamidation: A more environmentally safe process to proton conducting membrane for hydrogen HT-PEM fuel cell. Eur. Polym. J..

[20] Ponomarev I.I., Skupov K.M., Zhigalina O.M., Naumkin A.V., Modestov A.D., Basu V.G., Sufiyanova A.E., Razorenov D.Y., Ponomarev I.I. (2020). New Carbon Nanofiber Composite Materials Containing Lanthanides and Transition Metals Based on Electrospun Polyacrylonitrile for High Temperature Polymer Electrolyte Membrane Fuel Cell Cathodes. Polymers.

[21] Zhigalina V.G., Zhigalina O.M., Ponomarev I.I., Skupov K.M., Razorenov D.Y., Ponomarev I.I., Kiselev N.A., Leitinger G. (2017). Electron microscopy study of new composite materials based on electrospun carbon nanofibers. Crystengcomm.

[22] Ponomarev I.I., Skupov K.M., Naumkin A.V., Basu V.G., Zhigalina O.M., Razorenov D.Y., Ponomarev I.I., Volkova Y.A. (2019). Probing of complex carbon nanofiber paper as gas-diffusion electrode for high temperature polymer electrolyte membrane fuel cell. RSC Adv..

[23] Ponomarev I.I., Zhigalina O.M., Skupov K.M., Modestov A.D., Basu V.G., Sufiyanova A.E., Ponomarev I.I., Razorenov D.Y. (2019). Preparation and thermal treatment influence on Pt-decorated electrospun carbon nanofiber electrocatalysts. RSC Adv..

[24] Ponomarev I.I., Skupov K.M., Zhigalina O.M., Khmelenin D.N., Ponomarev I.I., Vtyurina E.S., Cherkovskiy E.N., Basu V.G., Modestov A.D. (2022). Deposition of Pt Nanoparticles by Ascorbic Acid on Composite Electrospun Polyacrylonitrile-Based Carbon Nanofiber for HT-PEM Fuel Cell Cathodes. Catalysts.

[25] Ponomarev I.I., Ponomarev I.I., Filatov I.Y., Filatov Y.N., Razorenov D.Y., Volkova Y.A., Zhigalina O.M., Zhigalina V.G., Grebenev V.V., Kiselev N.A. (2013). Design of electrodes based on a carbon nanofiber nonwoven material for the membrane electrode assembly of a polybenzimidazole-membrane fuel cell. Dokl. Phys. Chem..

[26] Vol’fkovich Y.M., Ponomarev I.I., Sosenkin V.E., Ponomarev I.I., Skupov K.M., Razorenov D.Y. (2019). A Porous Structure of Nanofiber Electrospun Polyacrylonitrile-Based Materials: A Standard Contact Porosimetry Study. Prot. Met. Phys. Chem. Surf..

[27] Ponomarev I., Skupov K.M., Razorenov D., Zhigalina V.G., Zhigalina O.M., Ponomarev I.I., Volkova Y.A., Kondratenko M., Bukalov S., Davydova E.S. (2016). Electrospun nanofiber pyropolymer electrodes for fuel cells on polybenzimidazole membranes. Russ. J. Electrochem..

[28] Ponomarev I.I., Filatov Y.N., Ponomarev I.I., Filatov I.Y., Razorenov D.Y., Skupov K.M., Zhigalina O.M., Zhigalina V.G. (2017). Electroforming of Nitrogen-Containing Polymers and Derived Nonfabric Nanofibre Carbon Materials. Fibre Chem..

[29] Skupov K.M., Ponomarev I., Razorenov D.Y., Zhigalina V.G., Zhigalina O.M., Ponomarev I.I., Volkova Y.A., Volfkovich Y.M., Sosenkin V.E. (2017). Carbon nanofiber paper cathode modification for higher performance of phosphoric acid fuel cells on polybenzimidazole membrane. Russ. J. Electrochem..

[30] Ponomarev I.I., Skupov K.M., Ponomarev I.I., Razorenov D.Y., Volkova Y.A., Basu V.G., Zhigalina O.M., Bukalov S.S., Volfkovich Y.M., Sosenkin V.E. (2019). New Gas-Diffusion Electrode Based on Heterocyclic Microporous Polymer PIM-1 for High-Temperature Polymer Electrolyte Membrane Fuel Cell. Russ. J. Electrochem..

[31] Skupov K.M., Ponomarev I.I., Vol’fkovich Y.M., Modestov A.D., Ponomarev I.I., Volkova Y.A., Razorenov D.Y., Sosenkin V.E. (2020). The Effect of the Stabilization and Carbonization Temperatures on the Properties of Microporous Carbon Nanofiber Cathodes for Fuel Cells on Polybenzimidazole Membrane. Polym. Sci. Ser. C.

[32] Skupov K.M., Ponomarev I.I., Razorenov D.Y., Zhigalina V.G., Zhigalina O.M., Ponomarev I.I., Volkova Y.A., Volfkovich Y.M., Sosenkin V.E. (2017). Carbon nanofiber paper electrodes based on heterocyclic polymers for high temperature polymer electrolyte membrane fuel cell. Macromol. Symp..

[33] Skupov K.M., Ponomarev I.I., Volfkovich Y.M., Sosenkin V.E., Ponomarev I.I., Volkova Y.A., Razorenov D.Y., Buyanovskaya A.G., Talanova V.N. (2020). Porous structure optimization of electrospun carbon materials. Russ. Chem. Bull..

[34] Inagaki M., Yang Y., Kang F. (2012). Carbon Nanofibers Prepared via Electrospinning. Adv. Mater..

[35] Tenchurin T.K., Krasheninnikov S.N., Orekhov A.S., Chvalun S.N., Shepelev A.D., Belousov S.L., Gulyaev A.I. (2014). Rheological Features of Fiber Spinning from Polyacrylonitrile Solutions in an Electric Field. Structure and Properties. Fibre Chem..

[36] Dong Z., Kennedy S.J., Wu Y. (2011). Electrospinning materials for energy-related applications and devices. J. Power Sources.

[37] Zhang W., Wang Y., Sun C. (2007). Characterization on oxidative stabilization of polyacrylonitrile nanofibers prepared by electrospinning. J. Polym. Res..

[38] Dubal S., Chavan S., Jadhav P. (2022). Oxidative Stabilization and Characterization of Electrospun Polyacrylonitrile Nanofiber Web on Different Substrates. J. Inst. Eng. India Ser. C.

[39] Yusof N., Ismail A.F. (2012). Post spinning and pyrolysis processes of polyacrylonitrile (PAN)-based carbon fiber and activated carbon fiber: A review. J. Anal. Appl. Pyrol..

[40] Zhang L., Aboagye A., Kelkar A., Lai C., Fong H. (2014). A review: Carbon nanofibers from electrospun polyacrylonitrile and their applications. J. Mater. Sci..

[41] Zhang H., Tan Y., Luo X.-D., Sun C.Y., Chen N. (2019). Polarization Effects of a Rayon and Polyacrylonitrile Based Graphite Felt for Iron-Chromium Redox Flow Batteries. ChemElectroChem.

[42] Zhang H., Chen N., Sun C., Luo X. (2020). Investigations on physicochemical properties and electrochemical performance of graphite felt and carbon felt for iron-chromium redox flow battery. Int. J. Energy Res..

[43] Low Z.-X., Budd P.M., McKeown N.B., Patterson D.A. (2018). Gas Permeation Properties, Physical Aging, and Its Mitigation in High Free Volume Glassy Polymers. Chem. Rev..

[44] Budd P.M., Ghanem B.S., Makhseed S., McKeown N.B., Msayib K.J., Tattershall C.E. (2004). Polymers of intrinsic microporosity (PIMs): Robust, solution-processable, organic nanoporous materials. Chem. Commun..

[45] Budd P.M., Msayib K.J., Tattershall C.E., Ghanem B.S., Reynolds K.J., McKeown N.B., Fritsch D. (2005). Gas separation membranes from polymers of intrinsic microporosity. J. Membr. Sci..

[46] Budd P.M., McKeown N.B., Ghanem B.S., Msayib K.J., Fritsch D., Starannikova L., Belov N., Sanfirova O., Yampolskii Y., Shantarovich V. (2008). Gas permeation parameters and other physicochemical properties of a polymer of intrinsic microporosity: Polybenzodioxane PIM-1. J. Membr. Sci..

[47] Wang L., Zhao Y., Fan B., Carta M., Malpass-Evans R., McKeown N.B., Marken F. (2020). Polymer of intrinsic microporosity (PIM) films and membranes in electrochemical energy storage and conversion: A mini-review. Electrochem. Commun..

[48] Thomas S., Pinnau I., Du N., Guiver M.D. (2009). Pure- and mixed-gas permeation properties of a microporous spirobisindane-based ladder polymer (PIM-1). J. Membr. Sci..

[49] Foster A.B., Beal J.L., Tamaddondar M., Luque-Alled J.M., Robertson B., Mathias M., Gorgojo P., Budd P.M. (2021). Importance of small loops within PIM-1 topology on gas separation selectivity in thin film composite membranes. J. Mater. Chem. A.

[50] Liu Y., Zhang J., Tan X. (2019). High Performance of PIM-1/ZIF-8 Composite Membranes for O_2_/N_2_ Separation. ACS Omega.

[51] Staiger C.L., Pas S.J., Hill A.J., Cornelius C.J. (2008). Gas Separation, Free Volume Distribution, and Physical Aging of a Highly Microporous Spirobisindane Polymer. Chem. Mater..

[52] Larsen G.S., Lin P., Hart K.E., Colina C.M. (2011). Molecular Simulations of PIM-1-like Polymers of Intrinsic Microporosity. Macromolecules.

[53] Song J., Du N., Dai Y., Robertson G.P., Guiver M.D., Thomas S., Pinnau I. (2008). Linear High Molecular Weight Ladder Polymers by Optimized Polycondensation of Tetrahydroxytetramethylspirobisindane and 1,4-Dicyanotetrafluorobenzene. Macromolecules.

[54] McKeown N.B. (2020). Polymers of Intrinsic Microporosity (PIMs). Polymer.

[55] Sizov V.E., Zefirov V.V., Volkova Y.A., Gusak D.I., Kharitonova E.P., Ponomarev I.I., Gallyamov M.O. (2022). Celgard/PIM-1 proton conducting composite membrane with reduced vanadium permeability. J. Appl. Polym. Sci..

[56] Mauritz K.A., Moore R.B. (2004). State of Understanding of Nafion. Chem. Rev..

[57] Karimi M.B., Mohammadi F., Hooshyari K. (2019). Recent approaches to improve Nafion performance for fuel cell applications: A review. Int. J. Hydrogen Energy.

[58] Peron J., Mani A., Zhao X., Edwards D., Adachi M., Soboleva T., Shi Z., Xie Z., Navessin T., Holdcroft S. (2010). Properties of Nafion^®^ NR-211 membranes for PEMFCs. J. Membr. Sci..

[59] De Almeida S.H., Kawano Y. (1999). Thermal Behavior of Nafion Membranes. J. Therm. Anal. Calorim..

[60] Schalenbach M., Hoefner T., Paciok P., Carmo M., Wiebke W., Stolten D. (2015). Gas Permeation through Nafion. Part 1: Measurements. J. Phys. Chem. C.

[61] Wainright J.S., Wang J.-T., Weng D., Savinell R.F., Litt M. (1995). Acid-Doped Polybenzimidazoles: A New Polymer Electrolyte. J. Electrochem. Soc..

[62] Bouchet R., Siebert E. (1999). Proton conduction in acid doped polybenzimidazole. Solid State Ionics.

[63] Mader J., Xiao L., Schmidt T.J., Benicewicz B.C., Scherer G.G. (2008). Polybenzimidazole/Acid Complexes as High-Temperature Membranes. Fuel Cells II. Advances in Polymer Science.

[64] Pu H., Meyer W.H., Wegner G. (2002). Proton transport in polybenzimidazole blended with H_3_PO_4_ or H_2_SO_4_. J. Polym. Sci. B Polym. Phys..

[65] Lobato J., Cañizares P., Rodrigo M.A., Linares J.J., Aguilar J.A. (2007). Improved polybenzimidazole films for H3PO4-doped PBI-based high temperature PEMFC. J. Membr. Sci..

[66] Bitter J.H., Tashvigh A.A. (2022). Recent Advances in Polybenzimidazole Membranes for Hydrogen Purification. Ind. Eng. Chem. Res..

[67] Ponomarev I.I., Ponomarev I.I., Petrovskii P.V., Volkova Y.A., Razorenov D.Y., Goryunova I.B., Starikova Z.A., Fomenkov A.I., Khokhlov A.R. (2010). Synthesis of N-phosphonoethylated cardo poly(benzimidazole) and testing of proton-conducting membranes made of it. Dokl. Chem..

[68] Ponomarev I.I., Blagodatskikh I.V., Muranov A.V., Volkova Y.A., Razorenov D.Y., Ponomarev I.I., Skupov K.M. (2016). Dimethyl sulfoxide as a green solvent for successful precipitative polyheterocyclization based on nucleophilic aromatic substitution, resulting in high molecular weight PIM-1. Mendeleev Commun..

[69] Ponomarev I.I., Blagodatskikh I.V., Muranov A.V., Volkova Y.A., Razorenov D.Y., Ponomarev I.I., Skupov K.M. (2020). Ultrasonic Activation of PIM-1 Synthesis and Properties of Polymers Obtained by Precipitation Polyheterocyclization in Dimethyl Sulfoxide. Polym. Sci. Ser. C.

[70] Schmidt T.J., Baurmeister J. (2008). Properties of high-temperature PEFC Celtec®-P 1000 MEAs in start/stop operation mode. J. Power Sources.

[71] Kondratenko M.S., Ponomarev I.I., Gallyamov M.O., Razorenov D.Y., Volkova Y.A., Kharitonova E.P., Khokhlov A.R. (2013). Novel composite Zr/PBI-O-PhT membranes for HT-PEFC applications. Beilstein J. Nanotechnol..

[72] Ponomarev I.I., Skupov K.M., Modestov A.D., Lysova A.A., Ponomarev I.I., Vtyurina E.S. (2022). Cardo polybenzimidazole (PBI-O-PhT) based membrane rein-forced with m-polybenzimidazole electrospun nanofiber mat for HT-PEM fuel cell applications. Membranes.

[73] Lysova A.A., Ponomarev I.I., Skupov K.M., Vtyurina E.S., Lysov K.A., Yaroslavtsev A.B. (2022). Effect of Organo-Silanes Structure on the Properties of Silane-Crosslinked Membranes Based on Cardo Polybenzimidazole PBI-O-PhT. Membranes.

[74] Rouquerol J., Rouquerol F., Sing K.S.W., Llewellyn P., Maurin G. (2012). Adsorption by Powders and Porous Solids: Principles, Methodology and Applications.

[75] Linares-Solano A., Stoeckli F. (2005). Commentary on the paper “On the adsorption affinity coefficient of carbon dioxide in microporous carbons” by E.S. Bickford et al. (Carbon 2004; 42: 1867–71). Carbon.

[76] Lysova A.A., Ponomarev I.I., Volkova Y.A., Ponomarev I.I., Yaroslavtsev A.B. (2018). Effect of Phosphorylation of Polybenzimidazole on Its Conductive Properties. Pet. Chem..

[77] Lozano-Castelló D., Cazorla-Amorós D., Linares-Solano A. (2004). Usefulness of CO_2_ adsorption at 273 K for the characterization of porous carbons. Carbon.

