# Supplementary material for: Proton-Conducting Polymer-Coated Carbon Nanofiber Mats for Pt-Anodes of High-Temperature Polymer-Electrolyte Membrane Fuel Cell
Source: Membranes (Basel). 2023 Apr 29;13(5):479. doi: 10.3390/membranes13050479 (PMC10224481; doi:10.3390/membranes13050479)
Supplement: Supplementary file 1 [file membranes-13-00479-s001.zip › membranes-2346411-supplementary.pdf]

Supporting Information

Proton-Conducting Polymer-Coated Carbon Nanofiber Mats for Pt-Anodes of High-Temperature Polymer-Electrolyte Membrane Fuel Cell

Kirill M. Skupov, Igor I. Ponomarev, Elizaveta S. Vtyurina, Yulia A. Volkova, Ivan I. Ponomarev, Olga M. Zhigalina, Dmitry N. Khmelenin, Evgeny N. Cherkovskiy, Alexander D. Modestov

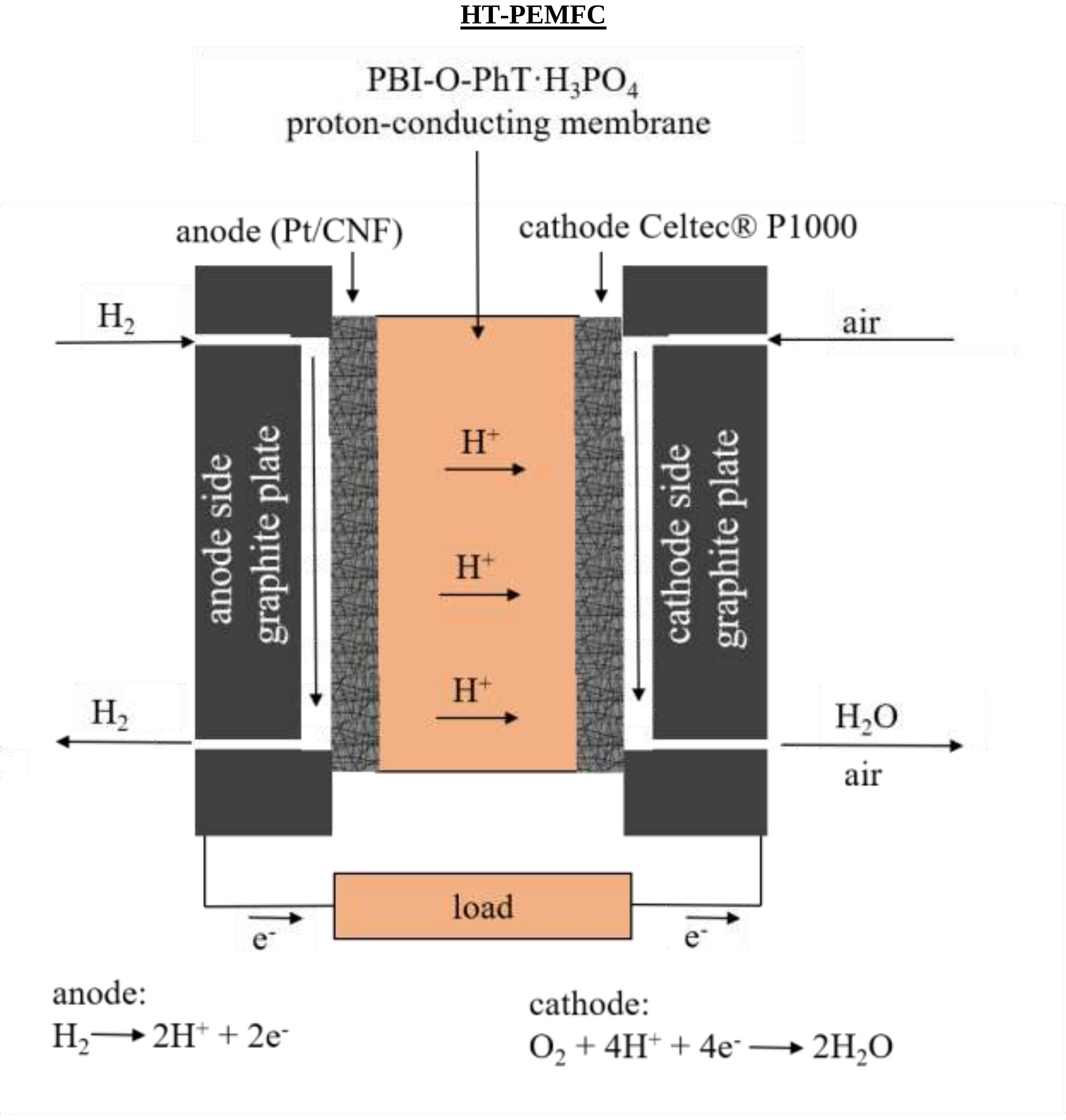

Figure S1. HT-PEMFC operation scheme.

EIS Nyquist plots

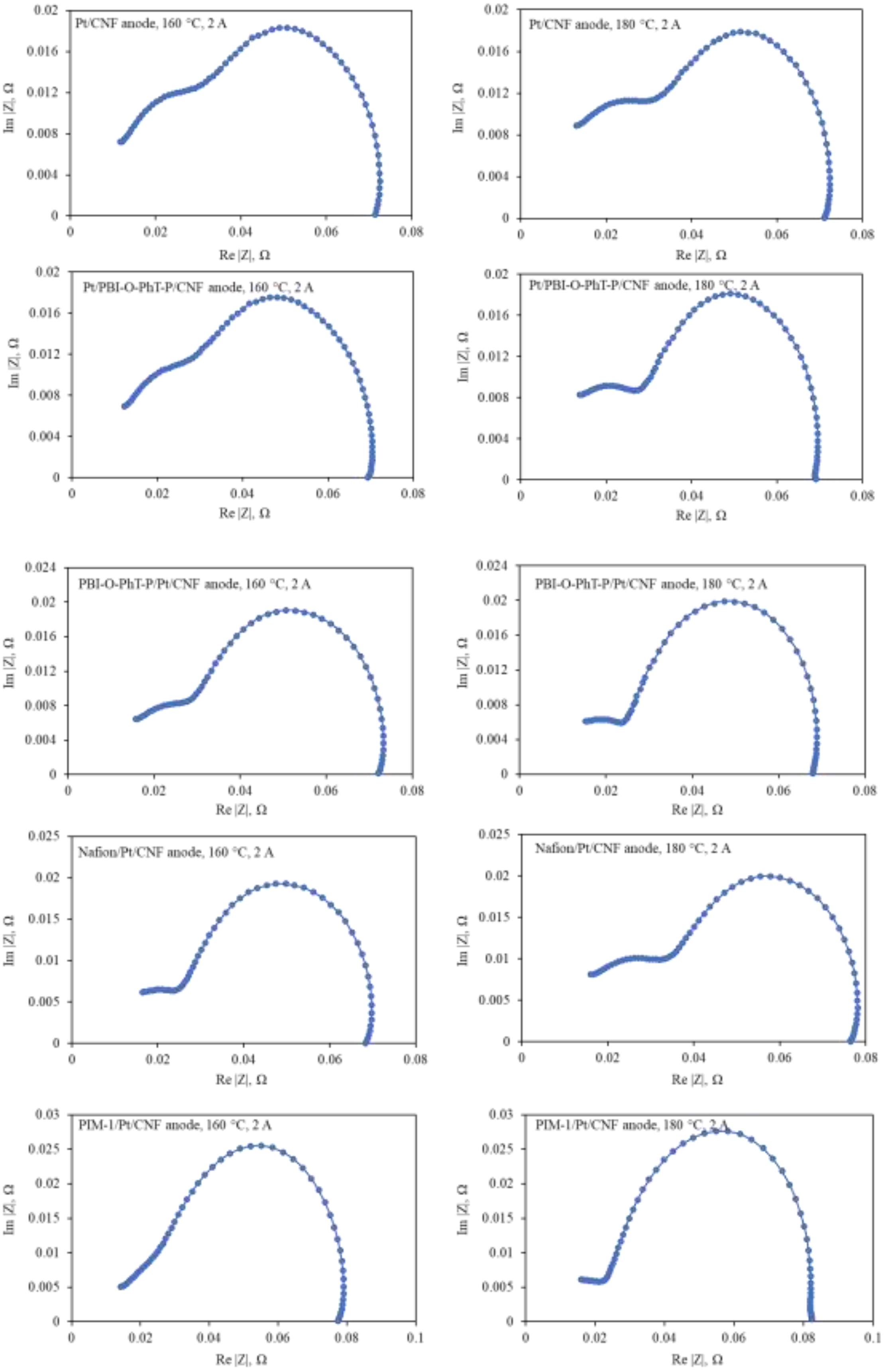

Figure S2. EIS Nyquist plots.
